# Genetic variation for grain nutritional profile and yield potential in sorghum and the possibility of selection for drought tolerance under irrigated conditions

**DOI:** 10.1186/s12864-023-09613-w

**Published:** 2023-09-02

**Authors:** Nasrein Mohamed Kamal, Yasir Serag Alnor Gorafi, Hisashi Tomemori, June-Sik Kim, Gamila Mohamed Idris Elhadi, Hisashi Tsujimoto

**Affiliations:** 1 Arid Land Research Center, Tottori University, Tottori, 680-0001 Japan; 2 Agricultural Research Corporation, PO Box 126, Wad Medani, Sudan; 3 International Platform for Dryland Research and Education, Tottori University, Tottori, Japan; 4 RIKEN Center for Sustainable Resource Science, Yokohama, 230-0045 Japan; 5 Institute of Plant Science and Resources, Okayama University, Kurashiki, 710-0046 Japan; 6 Faculty of Agriculture, Khartoum University, Kartoum North, PO Box 32, Khartoum, Sudan

**Keywords:** Africa, Asia, Diversity, Drought, GWAS, Seed nutrition, Sorghum

## Abstract

**Background:**

Increasing grain nutritional value in sorghum (*Sorghum bicolor*) is a paramount breeding objective, as is increasing drought resistance (DR), because sorghum is grown mainly in drought-prone areas. The genetic basis of grain nutritional traits remains largely unknown. Marker-assisted selection using significant loci identified through genome-wide association study (GWAS) shows potential for selecting desirable traits in crops. This study assessed natural variation available in sorghum accessions from around the globe to identify novel genes or genomic regions with potential for improving grain nutritional value, and to study associations between DR traits and grain weight and nutritional composition.

**Results:**

We dissected the genetic architecture of grain nutritional composition, protein content, thousand-kernel weight (TKW), and plant height (PH) in sorghum through GWAS of 163 unique African and Asian accessions under irrigated and post-flowering drought conditions. Several QTLs were detected. Some were significantly associated with DR, TKW, PH, protein, and Zn, Mn, and Ca contents. Genomic regions on chromosomes 1, 2, 4, 8, 9, and 10 were associated with TKW, nutritional, and DR traits; colocalization patterns of these markers indicate potential for simultaneous improvement of these traits. In African accessions, markers associated with TKW were mapped to six regions also associated with protein, Zn, Ca, Mn, Na, and DR, suggesting the potential for simultaneous selection for higher grain nutrition and TKW. Our results indicate that it may be possible to select for increased DR on the basis of grain nutrition and weight potential.

**Conclusions:**

This study provides a valuable resource for selecting landraces for use in plant breeding programs and for identifying loci that may contribute to grain nutrition and weight with the hope of producing cultivars that combine improved yield traits, nutrition, and DR.

**Supplementary Information:**

The online version contains supplementary material available at 10.1186/s12864-023-09613-w.

## Background

*Sorghum bicolor* (L.) Moench, one of the five main cereal crops globally [1], plays a vital role in global food security and is the staple food of billions of people [2]. Sorghum is a significant source of dietary energy, protein, and micronutrients for most sub-Saharan African populations [3]. Mann et al. [4] hypothesized that sorghum originated and was first domesticated in northeastern Africa. It subsequently spread to eastern and southern Africa, and by trade routes to Asia [5, 6]. Asia is considered the secondary center of sorghum diversity [7]. Landraces with high genetic and phenotypic diversity have been reported from Ethiopia [8, 9], Eritrea [10], Sudan [11], and India [12]. These are valuable sources of genetic variation that breeders can use to generate cultivars with higher productivity, nutrients, and climate resilience [13, 14].

Developing sorghum cultivars with enhanced grain nutritional quality is an important target for many breeding programs [15]. Sorghum is an essential source of iron and zinc and has higher mineral nutrition than rice and wheat [16]. Protein constitutes 12% of the grain on a dry-weight basis. Sorghum cultivars vary substantially in total protein, mineral nutrition, and amino acid profiles [17]. Biofortification of sorghum through genetic approaches and an increased intake of nutrition-rich sorghum grains could help to improve nutritional security in the developing world [18]. Understanding the natural variation and genetic architecture of grain nutritional traits in sorghum is the first step towards nutritional quality improvement through conventional and molecular breeding. However, modern sorghum cultivars have narrow genetic diversity for grain mineral profiles, thus identifying valuable alleles in landraces may be helpful for enhancing nutrition [15, 19, 20].

More than 90% of sorghum growing and 85% of processing (such as fermentation, cooking, soaking, etc.) are concentrated in warm semiarid areas [21]. Drought is one of the primary stresses, imposing a massive choke on sorghum growth, yield, and nutritional quality across its major cultivation areas [22–26]. Global production of sorghum, which is lower than that of other cereals such as wheat and rice, might be increased by exploiting the untapped potential present in its gene pool; cultivars represent < 15% of its genetic diversity (Fig. S1) [27]. Improvement of grain nutrition, yield, and drought tolerance or drought resistance (DR) in sorghum is challenging because the traits are genetically complex. A substantial interaction between drought tolerance traits and environment renders traits that are favorable in one environment, neutral or even negative in another [28, 29]. Breeding for DR thus requires laborious testing of a large number of lines in multi-environment trials and is time-consuming. Furthermore, dependency on a few sources of DR genes and alleles should be avoided. As the effects of climate change increase, there is a need to screen and identify novel germplasm harboring yield potential and quality traits that can be harnessed for adaptation to drought-prone areas.

Maintaining high productivity in crops exposed to environmental stresses depends on inherent mechanisms to avoid, adapt to, or tolerate stresses [30]. The grain mineral status of plants greatly affects their ability to adapt to adverse conditions. The effects of nutrient deficiencies include increased susceptibility to biotic stresses and decreased biomass accumulation, plant growth, and yield potential. Moreover, reduced tolerance to drought stress is associated with low nutrient uptake and accumulation in crop plants [30]. Grain minerals are essential for plant growth and their shortage rapidly leads to increased sensitivity to abiotic and biotic stresses [31].

Despite massive research efforts, the genetic mechanisms underlying DR and mineral contents remain poorly understood. As well, most recent studies on sorghum have investigated grain yield, quality, or stress tolerance–related traits in isolation [22–26, 30, 31]. Exploring available germplasm diversity for all of these traits would facilitate the development of crop cultivars that combine stress tolerance, high yield potential, and nutritional value.

We examined the mineral or nutritional content, thousand kernel weight (TKW), protein content, and plant height in 163 germplasm accessions grown under normal conditions and three DR traits in plants grown under post-flowering drought conditions. We investigated the genetic control of these traits and their associations using a genome-wide association study (GWAS) approach. We also established a method for screening cultivars for DR on the basis of their grain weight and nutritional profile under normal conditions without the need for complicated field testing.

To the best of our knowledge, this is the first attempt to explore the phenotypic and genotypic associations between sorghum grain weight, nutritional potential, and DR traits intended to identify valuable germplasm or to establish screening methods to develop drought-tolerant cultivars with high yield potential in terms of PH and TKW and nutrition without field testing. It provides a valuable resource for elucidating loci associated with traits of interest that may be useful in understanding mechanisms that contribute to grain nutrition and weight and in future breeding programs that seek to produce sorghum cultivars that combine high grain yield potential, nutrition, and DR.

## Results

### Population structure and genetic diversity

Allele frequencies and polymorphic information content (PIC) values are summarized in Supplementary Table 1. The average degree of polymorphism was 0.02.

**Fig. 1:**
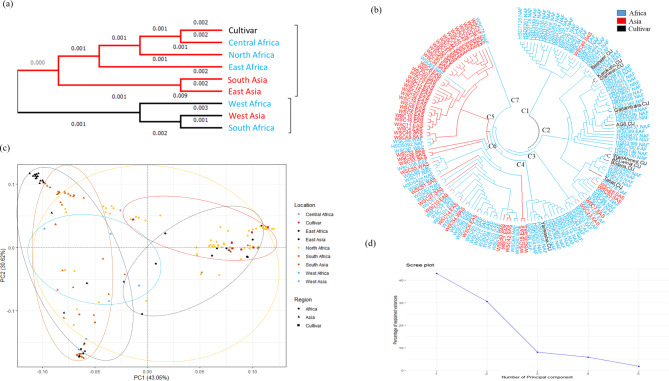
(**a**) Cluster analysis showing significant associations among the cultivars and accessions from eight African and Asian regions. (**b**) Neighbor-joining phylogenetic tree of the 163 sorghum accessions. (**c**) Principal component analysis. (**d**) Scree plot

Cluster analysis explore differences and similarities between geographic groups showed that the accessions formed nine groups, in two separate clusters. The first cluster contained the Sudanese cultivars, the Central, North, and East African accessions, and a sub-cluster of accessions from South and East Asia (Fig. 1a). Interestingly, the second major cluster contained the West Asian, South African, and West African accessions (Fig. 1a). The neighbor-joining phylogenetic tree showed seven clusters (C1–C7; Fig. 1b). Clusters 1, 2, 3, and 4 consisted primarily of improved cultivars and accessions of African origin. At least 80% of accessions in Clusters 1, 2, and 4 were landraces from North Africa. Cluster 5 was composed of landraces mostly of Asian origin (Fig. 1b). Cluster 6 contained a mixture of African and Asian accessions.

We also performed principal component analysis (PCA) to investigate the relationships among accessions. PC1 and PC2 captured ~ 73.7% of the genetic variation (Fig. 1c, d). Integrating the nine regions of origin into the PCA biplots of PC1 vs. PC2 revealed six clusters, following the same trend as in the geographic origin cluster (Fig. 1c).

Analysis of molecular variance (Table 1) showed that variation among groups was 10%, while variation within groups was 90%.

### Phenotypic variation

**Fig. 2:**
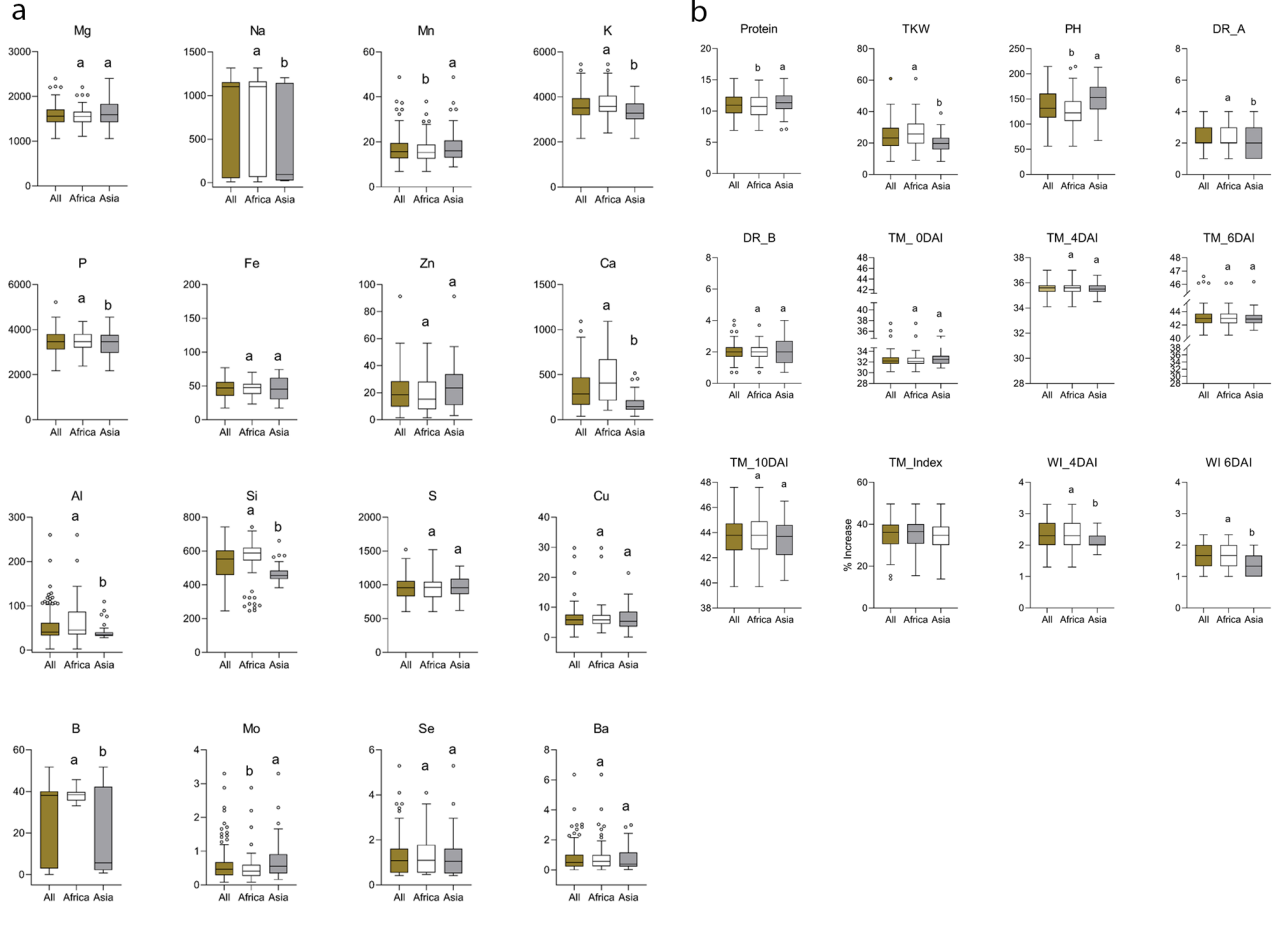
Box plots showing the variation within all, African, and Asian accessions for (**a**) 16 grain elements (mg kg^− 1^), and (**b**) protein content (%), thousand kernel weight (TKW/g), plant height (PH/cm), drought resistance score at 4 (DR_A) and 6 days after irrigation (DR_B), leaf temperature at 0, 4, 6, and 10 days after irrigation (TM_0DAI, TM_4DAI, TM_6DAI, TM_10DAI), % increase of leaf temperature after 10 days of drought (TM_Index), and withering index at 4 and 6 days after irrigation (WI 4DAI, WI 6DAI). Location means followed by different letters are significantly different at P < 0.05, Tukey test

The 163 Asian and African accessions showed a wide range of phenotypic variation. In general, seed mineral contents had the highest phenotypic variation, with Na, Mo, Fe, and Cu, Mg and P displaying higher variation (Tables S2–S7; Fig. 2a).

**Table 1:** Analysis of molecular variance of all accessions using 8938 SNPs as markers

Source of variation	df	Sum of squares	Mean of squares	Estimate variance	Percentage of variance
Among population	8	2389.2	298.65	12.021	10%
Within population	151	16966.4	112.361	112.361	90%
Total	159	19355.6		124.382	
Pairwise population (PhiPT)	0.097				

*Probability, *P* (rand ≥ data), for PhiPT is based on standard permutation across the full data set

Average values for accessions from each region are shown in Tables S2–7. African and Asian accessions differed significantly in 15 out of the 28 traits studied (Fig. 2a, b). Plant height (PH) ranged from 56.2 to 214.6 cm (mean = 136.5 cm), TKW from 8.2 to 61.1 g (mean = 24.5 g), and protein content from 6.9 to 15.2% (mean = 10.9%). Accessions from East Asia had the lowest TKW values and those from North Africa had the highest. West African and South Asian accessions had the greatest average height, whereas those from West Asia were shortest, on average (Tables S2–S7). West Asian accessions had the lowest average protein content, whereas Central African and South Asian accessions had the highest (Tables S2–S7).

African accessions had higher seed mineral content than Asian accessions. In all accessions, macroelement contents were 2173.0–5220.6 mg/kg P, 2160.9–5452.0 mg/kg K, 1055.2–2402.4 mg/kg Mg, 36.5–1091.5 mg/kg Ca, and 9.3–1315.7 mg/kg Na. Microelement contents were 17.3–77.0 mg/kg Fe, 6.9–48.7 Mn, 2.8–85.0 mg/kg Zn, 1.2–29.7 mg/kg Cu, 0.1–3.3 mg/kg Mo, 0.01–0.80 mg/kg Co, and 246.4–742.6 mg/kg Si.

Drought resistance at 4 days after drought (DR_A), withering at 4 days after drought (WI_4DAI), and withering at 6 days after drought (WI_6DAI) were significantly higher in African than in Asian accessions (Fig. 2b). After 10 days of drought, the pots were re-irrigated, and 41 accessions recovered, 26 from Asia and 15 from Africa (Fig. S2). The DR score was > 3 for 10 and 2 Asian accessions 3 and 5 weeks after re-irrigation (WAI), respectively. In the African accessions, DR was > 3 in 3 and 1 accessions 3 and 5 WAI, respectively (Fig. S2). Interestingly, Asian accessions with high DR scores had higher protein values and lower TKW than the African accessions with high DR (Table S8).

### Pearson’s correlation, phenotypic-based hierarchical clustering, and PCA across African, Asian, and all accessions

**Fig. 3:**
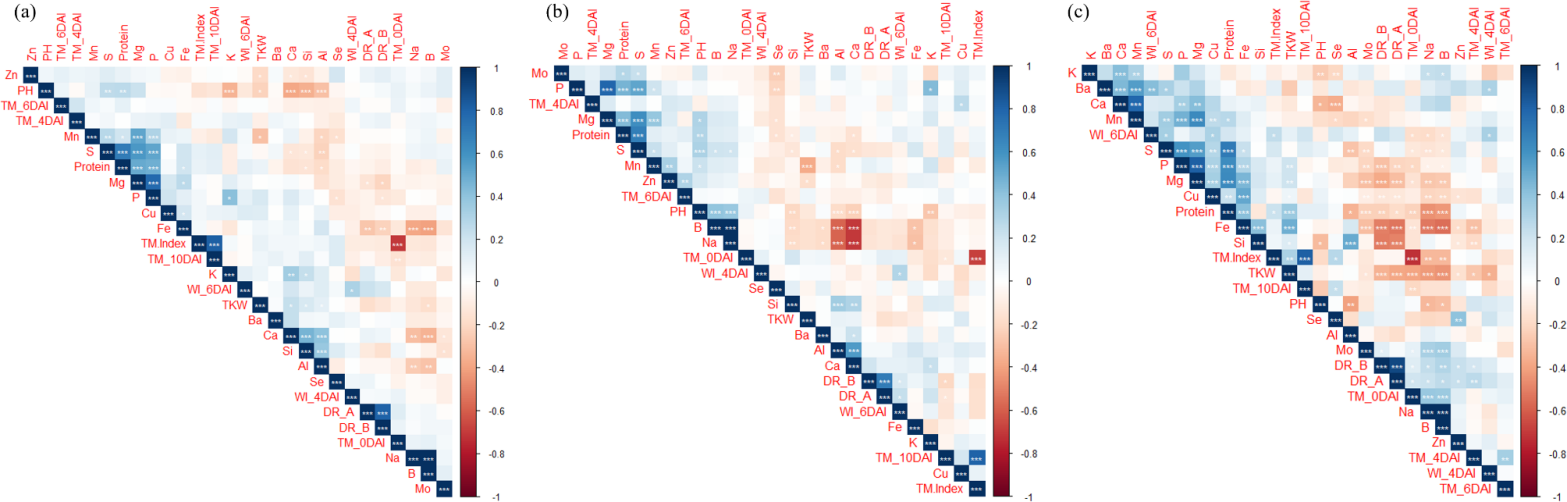
Pearson’s correlation analysis of 16 grain elements, protein content, thousand kernel weight (TKW), plant height (PH), drought resistance score at 4 (DR_A) and 6 days after irrigation (DR_B), leaf temperature at 0, 4, 6, and 10 days after irrigation (TM_0DAI, TM_4DAI, TM_6DAI, TM_10DAI), % increase of leaf temperature after 10 days of drought (TM_Index), and withering index at 4 and 6 days after irrigation (WI 4DAI, WI 6DAI) in (**a**) all, (**b**) African, and (**c**) Asian accessions. *, ** and ***: indicated significant levels at < 0.05, < 0.01 and < 0.001, respectively

Among all accessions, protein content correlated positively with Mg (0.50**), P (0.48***), and Fe (0.20*) contents (Fig. 3a). TKW correlated negatively with B (-0.18*), Zn (-0.19*), PH (-0.23**) and Mn (-0.29**) and positively with Ca (0.23**), Si (0.17*) and Al (0.19*). Interestingly, the leaf temperature index (TM_Index) was negatively correlated with the leaf temperature before drought (TM_0 DAI) (-0.70**) (Fig. 3a). The African accessions had negative correlations between TKW and Mn (-0.37**) and Zn (-0.18*) content, Positive correlations between PH and B (0.39**), Na (0.38**) and Mg (0.30**), and negative correlations between PH and Si (-0.20*), Al (-0.20*), Ca (-0.23*) and K (-0.27*) contents (Fig. 3b). Mg content correlated positively with protein content (0.43**), PH (0.30*), S (0.62**) and Mn (0.38***). In the Asian accessions, both DR_A and DR_B had negative correlations with, Mg (-0.3* and − 0.40**, respectively), Cu (-0.37** and − 0.39**, respectively), Fe (-0.53** and − 0.0.50**, respectively), TKW (-0.38** and − 0.35*, respectively) and Protein content (-0.18* and − 0.23**, respectively). Interestingly, a positive correlation was found among TKW, protein content (0.37**), Fe (0.49**), and TM_Index (0.31*) (Fig. 3c).

**Fig. 4:**
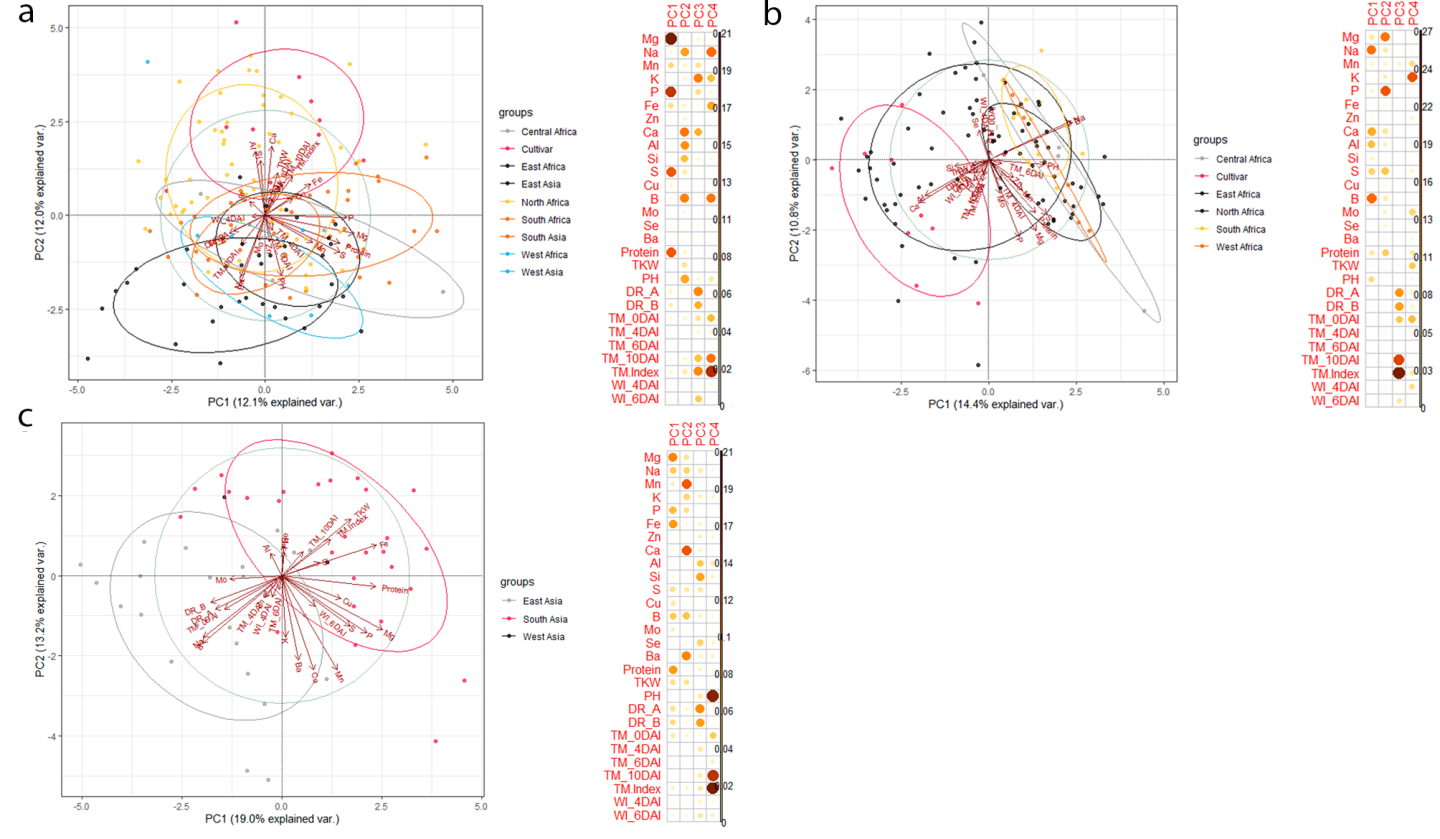
Principal component analysis (PCA) of 16 grain elements, protein content, thousand kernel weight (TKW), plant height (PH), drought resistance score at 4 (DR_A) and 6 days after irrigation (DR_B), leaf temperature at 0, 4, 6, and 10 days after irrigation (TM_0DAI, TM_4DAI, TM_6DAI, TM_10DAI), % increase of leaf temperature after 10 days of drought (TM_Index), and withering index at 4 and 6 days after irrigation (WI 4DAI, WI 6DAI) in (**a**) all, (**b**) African, and (**c**) Asian accessions

We conducted PCA and hierarchical cluster analysis of phenotypic and mineral traits to explore associations between traits and regions of origin. PC1 and PC2 accounted for 24.1% of the variation in all populations, 25.2% in African, and 32.2% in Asian (Fig. 4a, b, c). The all-accessions PCA plot showed overlap between accessions from Asia and Africa. However, accessions from some regions had distinct variation. Contents of Mg, P, S, Na, B, Ca, Al, PH and protein contributed most to accessions’ distribution patterns, followed by DR_A and B, K and TM_Index (Fig. 4a).

Among African accessions, North African accessions had a continuous distribution across PC1 and PC2, and they made the greatest contribution to the variation in the PCA (Fig. 4b). South, Central, and West African accessions were clustered together. Of the Asian accessions, South Asia and East Asia showed little overlap (Fig. 4c).

The hierarchical cluster analysis indicated that the 163 sorghum accessions formed two main clusters, each with two sub-clusters (a total of four sub-clusters) based on trait similarity (Fig. S3). The traits had two main clusters; the first cluster contained only the leaf temperature traits whereas the second cluster with remaining traits arranged in four sub-clusters. Sub-cluster 1 had only Na and B, Sub-cluster 2 had Mg, P, PH, S and protein content, sub-cluster 3 had only DR_A and B, and subcluster 4 included the remaining traits. The clustering pattern indicated significant variability among the landraces. Both African and Asian accessions were distributed in the two main groups without any clear geographical pattern. The Sudanese cultivars were assigned to Group 2, with the highest TKW, protein content, and some essential elements (Fig. S3). To summarize the phenotypic traits results and identify the uniqueness of each germplasm group we constructed a VIP score plot (which is based on PLSD analysis) that clearly elucidated the unique traits of each germplasm group. For example, cultivars, North and West Africa groups were superior in Ca contents, besides DR_A and B (Fig. S4). South Asia accessions were very rich in Mg, Fe, S, protein content, TKW and TM_Index (Fig. S4).

### Genome-wide marker-trait associations

**Fig. 5:**
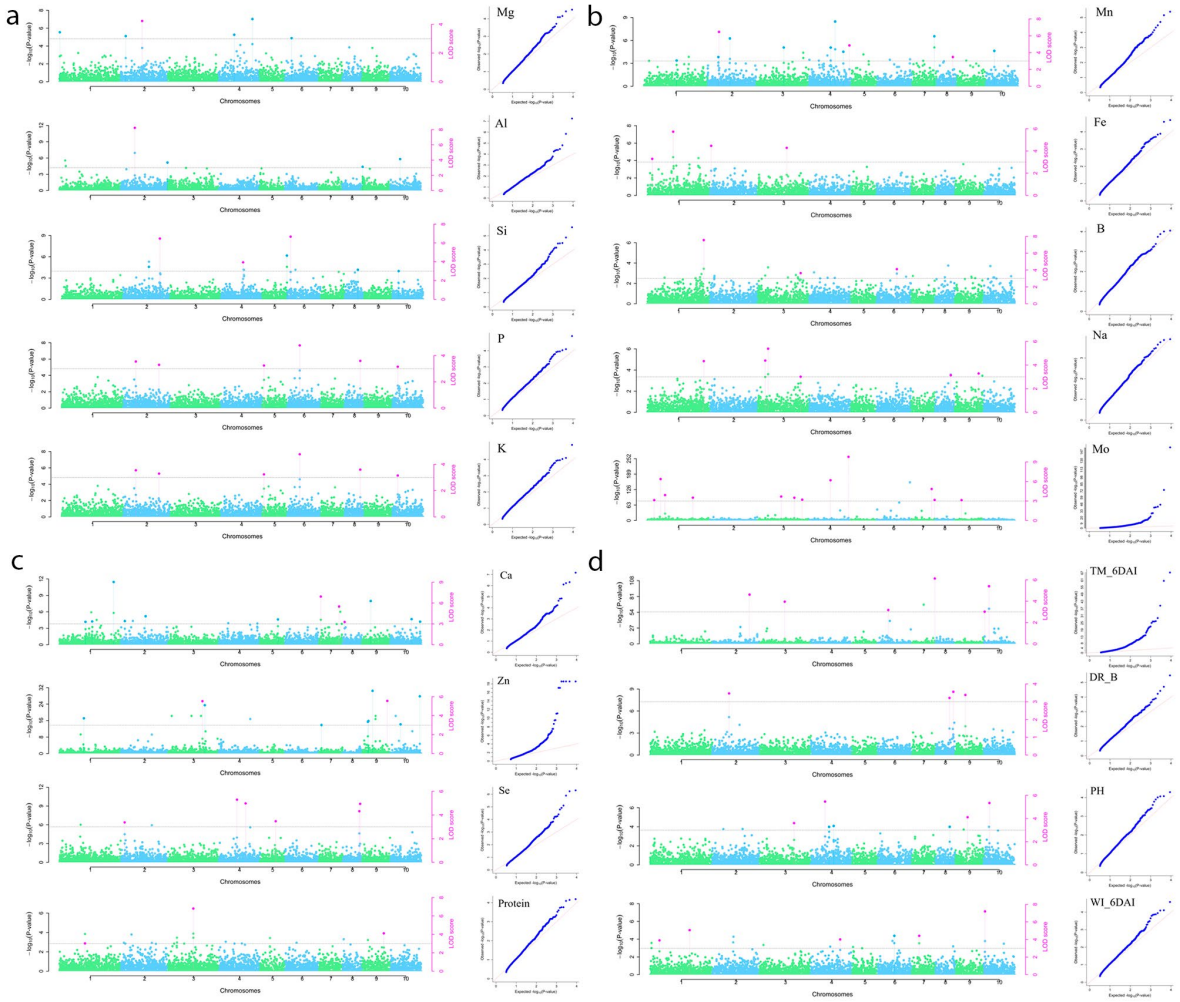
Manhattan plots and quantile–quantile plots of (**a**) Mg, Al, Si, P, and K grain concentrations, (**b**) Mn, Fe, B, Na, and Mo grain concentrations, (**c**) Ca, Zn, Se grain concentrations, and protein content and (**d**) leaf temperature at 6 days after drought (TM_6DAI), drought resistance at 6 days after irrigation (DR_B), plant height (PH), and withering index at 6 days after irrigation (WI_6DAI) in all accessions

We performed a genome-wide association study (GWAS) of all accessions and of African accessions (105), but not of Asian accessions, as their numbers were low (< 100). We used two algorithms, the multi-locus random-SNP-effect mixed linear model (mrMLM) and the FAST multi-locus random-SNP-effect mixed linear model (FASTmrMLM) [32]. GWAS returned 118 (mrMLM) and 89 (FASTmrMLM) significant SNP–trait associations, revealed by 18 traits identified in all accessions and 19 in African accessions. Quantile–quantile plots (Fig. 5a–d) showed good agreement between the expected and observed log_10_(*P*) values, as reflected by low scores following the null hypothesis line. Particularly for Al, P, and TKW marker trait associations (MATs), mrMLM produced higher -log_10_(*P*) values than expected yet showed greater statistical power, as reflected by the higher number of significant associations detected (Tables S9 and S10).

The complete list of markers significantly associated with phenotypes is provided in tables S9 and S10 report; the GWAS output is shown in Manhattan plots (Fig. 5a–d). In all accessions, chromosomes (Chr.) 1 and 2 had the highest number of associations (19 and 20, respectively). In African accessions, Chrs. 2 and 3 had the highest number of associations (18 and 11, respectively). Ca, Mo, and Mn had the highest number of associations in all accessions, whereas Ca, DR_B, and TKW recorded the highest number of associated SNPs in the African accessions (Tables S9 and S10).

In all accessions, the MTAs for the DR traits DR_B and WI_6DAI (10 MTAs) were distributed on Chrs. 1, 2, 4, 6, 7, 8, 9, and 10; most were identified by both mrMLM and FASTmrMLM. In African accessions, the drought-related traits were associated with 14 SNPs on all chromosomes except Chrs. 4 and 7 (Tables S9 and S10). In all accessions, six markers exhibited pleiotropic effects (rs = 2,658,844 on Chr. 2, Mg-P; rs = 19,031,410 on Chr. 10, Al-Zn; rs = 41,825,533 on Chr. 8, K-Mn; rs = 2,665,434 on Chr. 1, B-Na; rs = 2,655,071 on Chr. 3, B-Na; rs = 4,375,543 on Chr. 1, Ca-TKW; Table S9). In African accessions, only one marker (rs = 1,916,115 on Chr. 9) had a pleiotropic effect on B and Na (Table S10).

**Fig. 6:**
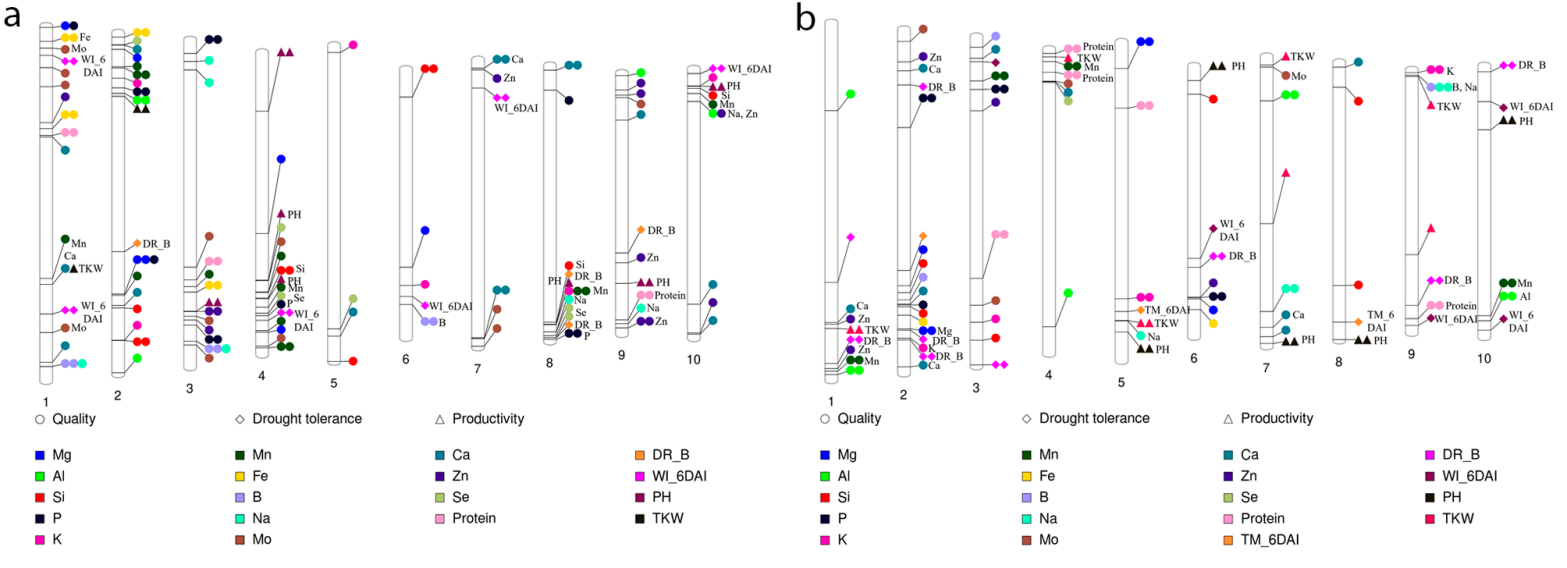
Significant marker-trait associations (MTAs) for 13 grain elements, protein content, thousand kernel weight (TKW), plant height (PH), drought resistance at 4 and 6 days after irrigation (DR_A, DR_B), leaf temperature at 4 and 6 days after drought (TM_4DAI, TM_6DAI), and withering index at 4 and 6 days after irrigation (WI_4DAI, WI_6DAI) in (**a**) all and (**b**) African sorghum accessions

In all accessions, markers related to minerals, WI_6DAI, protein content, and TKW co-located in the region from 0.379 to 58.557 Mbp on Chr. 1. Markers related to WI_6DAI were co-located with markers related to PH and P on Chr. 4 (51.656–60.751 Mbp), with markers related to PH and Zn on Chr. 10 (1.1327–3.9496 Mbp), and with markers related to Zn on Chr. 7 (2.4337–6.5319 Mbp). DR_B-related markers co-located with markers related to PH, Zn, and protein content on Chr. 9 (40.8101–55.9349 Mbp), and with markers related to minerals and PH on Chr. 8 (58.1326–62.1117 Mbp) (Fig. 6a). In African accessions, markers associated with different traits were colocalized on all chromosomes except Chrs. 4, 7, and 10; for instance, markers related to DR_B, TKW, Ca, and Zn co-localized in the region from 58.4655 to 68.8211 Mbp on Chr. 1, and those related to DR_B, WI_6DAI, TKW, and protein content were co-localized on Chr. 9 in the region from 41.6150 to 57.4313 Mbp (Fig. 6b).

In all accessions, 14 markers had a significant effect (*R*^2^ ≥ 15%), as defined by Habyarimana et al. [33]; these markers explained > 15% of the phenotypic variability, 6 in drought-related traits, protein content, Mg, and Zn (Table S9). In African accessions, 31 markers had significant effects (explaining > 15% of variation); 17 were associated with DR-related traits, TKW, PH, protein content, Zn, Fe, and Ca (Table S10).

### Pairwise statistical association among significant SNPs

In all accessions, several SNPs of highly significant markers, frequent to a common haplotype, were identified; Fig. S5 reports pairwise Pearson’s coefficients (*r*) for all significant markers identified by GWAS on all chromosomes. The WI_6 DAI SNPs (moderate drought; 6 days from irrigation) associated positively with P, Mn, Na, Fe, Mo, B, and Se and negatively with TKW, whereas DR_B (severe drought; 10 days from irrigation) SNPs associated positively with TKW and Zn and negatively with protein content and eight minerals. Protein content SNPs associated positively with Ca, and PH SNPs associated positively with TM_6 DAI, P and Mn (Fig. S5).

## Discussion

Sorghum is drought-tolerant and a rich source of minerals, vitamins, protein, and carbohydrates that are essential for human (grain) and animal (biomass) consumption. Knowledge of its nutritional, yield traits (PH and TKW), and DR genetic diversity and their associations would benefit breeding programs for improvement of yield and quality traits in drought-prone areas. We evaluated traits related to yield potential (TKW, PH) and seed quality or nutrition (16 macro- and micro-elements and protein content) in plants grown without drought, as well as DR traits (WI, leaf TM, and DR scores A and B) in plants grown under post-flowering drought. We explored the genetic diversity of these traits in order to identify landraces for use in breeding better sorghum cultivars and to assess the feasibility of predicting DR in plants grown under controlled conditions by evaluating their mineral and potential yield profiles.

## Genetic diversity and population structure

Genetic studies have investigated polymorphism in sorghum genotypes and assessed the extent of genetic variability. However, few studies have explored the genetic diversity in sorghum nutrition and yield traits values. This study provides a comprehensive and detailed evaluation of genetic diversity in sorghum through analysis of collections with known geographic origins by using high-throughput diversity array technology (DArT) markers across the entire genome.

The sorghum accessions used represent an excellent genetic diversity resource covering a wide range of geographical and ecological zones. Accessions showed the greatest similarity in SNP markers to others from the same geographic region (Fig. 1). The relationship between the African and Asian accessions indicates that sorghum was introduced from Africa into Asia, and the differentiation between them suggests further independent domestication or selection in Asia [6]. On the other hand, clustering of some Asian accessions with some African accessions suggests a recent introduction from Africa into Asia. Generally, our results indicate that the South and East Asian accessions may have been introduced from East, North, or Central Africa, whereas the West Asian accessions were introduced from South and West Africa [7, 34].

The PCA plots and hierarchical clustering indicate that the North African (center of origin) accessions are the most divergent from the other accessions; accessions from Africa appear to have little within-group diversity, whereas the Asian accessions have greater within-group diversity (Fig. 1a–d). Sorghum, both cultivated and wild, has high genetic diversity, with the greatest variation in Ethiopia and Sudan (northeast Africa), where it likely originated [35]. In addition, PCA revealed that some North and East African accessions fall into two distinct groups, whereas the Asian accessions fall into three groups; both African and Asian accessions have high levels of genetic variation. These results may be due to variation between geographic regions of Asia and because germplasm from Asia is relatively untapped (Fig. S1a).

According to AMOVA, 90% of the total variation occurred within and 10% occurred among populations (region of origins). The higher variation within regions of origin suggests lower human selection pressure on landraces than on sorghum grown elsewhere in these regions; strict selection by breeders for crop improvement might have contributed to reduce within-accession variation. As genetic diversity indicates potential to adapt to environmental change [36], sorghum landraces from these regions may serve as a potential source of genes for resistance to stresses. High levels of within-population variability among the sorghum landrace collections indicate high germplasm diversity and trait-based genetic novelty, which could contribute to improving the studied traits and adaptability [37]. On the other hand, the low variation among populations indicates high levels of gene flow between populations (region of origins) in different countries. Gene flow encompasses several mechanisms of gene exchange among populations, including the movement of gametes, zygotes, individuals, or groups of individuals from one place to another [38]. In this context, patterns of seed exchange among communities could be the main factor driving the similarities among accessions from different regions.

### Variation in seed traits

We studied the phenotypic variation of 16 grain mineral contents and several phenotypic traits. We could not confirm any clear relationship between molecular and morphological traits. This could be attributed to the relatively small sample size and the fact that phenotypic traits are influenced by environment. Ayana et al. [39] found no genetic relationship with agro-morphological traits, and no correlation between molecular markers and morphological traits. Similarly, Dahlberg et al. [40] reported a poor relationship between molecular markers and agronomic descriptors. As environment influences morphological characteristics, changes in environmental factors are far more likely to alter morphological characteristics than the underlying genetic structure. Our results are supported by other findings [41, 42].

The control of macro- and micronutrient homeostasis in plants has been extensively studied; however, the loci that control natural ionomic variations in the grain of sorghum are still largely undetermined. The discovery of genes or genomic regions associated with grain nutritional content and TKW may accelerate the development of new biofortified cultivars. Accessions showed a broad range of mineral concentrations (Tables S3–S5; Fig. 2). African accessions had higher mineral composition than Asian accessions overall, especially in the Central, East, and West Africa regions. Among Asian accessions, those from East and South Asia were the richest in mineral and protein contents. The ranges and means of mineral contents were similar to or higher than those reported by Kumar et al. [43]; accessions from Africa and Asia had similarly high ranges of Fe and Zn (> 60 mg kg^− 1^; Fig. 2). Ng’uni et al. [19] reported that grain Fe concentration ranged from 2.8 to 6.3 mg/100 g and grain Zn from 2.3 to 5.5 mg/100 g in South African sorghum cultivars.

Mg, Fe, S, and Cu had relatively low levels of variation (Fig. 2), indicating that homeostasis of these elements is under relatively tight regulation. Previous research has shown that plants have evolved regulatory mechanisms to control the internal fluctuation of essential nutrients so as to maintain their concentrations within narrow ranges for optimal growth, development, and seed production [44, 45]. On the other hand, elements that varied significantly among or within regions, such as Na, B, Mo, and Ca, are likely under less regulatory pressure and therefore likely have simpler control mechanisms. Control mechanisms can differ among genotypes, within a single plant, and among geographical regions [46]. Because of this regulatory variability, enhancing the micronutrient density of edible plant components through the manipulation of physiological processes is environment-specific [46]. Elements regulatory variability may also be attributed to their essential role as stress response factors, e.g., Ca is a key messenger for coordinating the activity of eukaryotic cells. Ca^2+^ acts as a control ion in a wide variety of plant cells, and Ca signaling potential is linked to the ability of cells to maintain high concentration gradients [47]. Another factor that might be responsible for the wide variation in micronutrient contents could be differences in translocation rates between different soil and climate conditions. Different genotypes may exhibit different abilities to take up soil nutrients [48]. Germplasm lines with high mineral content and high yield traits (PH and TKW) capacity could be used in recombination breeding to improve sorghum’s nutritional value.

Protein content varied widely (6–15%). Asian accessions had the highest content. Both environment and genotype affect protein content [49]. African accessions tended to have a higher C/N ratio (i.e., less protein content and more carbohydrates). Temperature and precipitation were essential in the accession’s adaptability to TKW determination, protein contents, and DR. The low protein content of African sorghum may be due to the climate or the selection pressure against grain quality, grain weight, or DR is independent.

### Variation in productivity traits (PH and TKW)

The yield parameters of average PH and TKW tended to be higher in African accessions, especially from North and West Africa; grain weight is a crucial determinant of yield-related traits in cereals. Accessions from those areas are promising sources of genetic material for high TKW and mineral concentration. Among sorghum cultivars, those of *S. bicolor* retain the most similarity to wild ancestors, and high protein, mineral contents, and TKW may have been inadvertently counter selected during cereal domestication when high starch and grain yield were selected. Furthermore, human selection in these areas for different food or feed uses influenced the patterns of grain composition distribution among genetic groups; porridges from different regions require different grain compositions. The significant correlations between PH and grain yield and TKW were reported in sorghum by Kante [50], which showed yield superiority of the tall hybrids over the well-adapted local varieties of West and Central Africa. Accordingly, our result is consistent with that of Kante and Adedugba [50, 51].

Our diverse association panel’s protein, TKW, PH and mineral content ranges may help improve sorghum yield potential and quality. Furthermore, adaptation to different environments drives grain composition differences between genetic groups. Evidence of this adaptation was recently found in tannins in sorghum grain when a variant of the *Tannin 1* gene, which controls the presence of tannins in the grain, was found to be correlated with several bioclimatic factors [52].

### Phenotypic trait correlations

Correlations between traits are of great importance for successful selection in breeding programs. Here, protein content was positively correlated with TKW, Cu, Mg, P, S, and Zn. These results suggest that the best strategy may be to combine selection for micronutrients, protein, and TKW in Asian accessions in a single agronomic background. However, for cultivars with improved micronutrient profiles to benefit human nutrition, the cultivars must carry farmer-preferred grain traits such as earliness, seed size, and color [19]. Other studies have found negative correlations between grain yield traits (PH and TKW) and desirable traits, such as Fe and Zn in maize [53], and sorghum [18]. In African accessions, Mn and Zn were negatively correlated with TKW and showed no relationship with other elements, whereas in Asian accessions, P, Mg, Cu, Fe and Si were positively correlated with TKW (Fig. 3b, c). The absence of correlations between TKW and grain element concentrations in African accessions likely reflects that increasing TKW had no effect on elemental concentrations. Significant correlations among nutrients suggest that their concentrations in sorghum landraces can be simultaneously improved [54]. Associations between traits and their contribution to diversity can be validated by multivariate analysis. High-yielding germplasm lines in terms of PH and TKW with improved micronutrient levels and DR were grouped in Cluster 2 (Fig. S3). Selection and crossing of genotypes from different clusters would help bring together genes favorable for yield and quality traits to breed tailor-made cultivars [55].

### Breeding drought-tolerant sorghum by selecting for yield and nutritional potential

The wide phenotypic variation in minerals, protein content, and TKW indicates that our accessions panel is well suited to dissecting the genetic basis of high nutritional value and TKW in sorghum.

We found no correlations between TKW, PH, protein, and most elements, except for the negative correlation of Mn and B and the positive correlation of Al, Se, and Ca. However, PH QTLs co-located with QTLs for Mn, P, Na, Si, Se, Mo, and DR on Chrs. 4 and 10 and WI_6DAI on Chr. 8 (Fig. 6a). Co-localization of TKW, PH, Na, and Tm_6DAI was found only in African accessions on Chr. 5 (Fig. 6a). This suggests that improved grain elements, PH, and TKW can be selected for simultaneously. In African accessions, Zn, Mn, and Ca were mostly co-localized with TKW and PH on Chrs. 1, 4, and 7. A previous study identified significant MTAs of biomass-related traits on Chrs. 7 and 9 [56].

Interestingly, QTLs for Zn and Ca were co-localized with those for DR_B on Chrs. 1 and 2 and with those for Al, Mn, K, Mg, and Fe (Fig. 6b). In theory, a taller plant will have more biomass and hence can accumulate more minerals during vegetative growth, providing a significant source for remobilization from leaves when they senesce at grain filling, especially during drought stress [23, 57, 58]. On Chr. 1, a locus with a major effect (16.2%) on dry mass was co-localized with a zinc-finger homeodomain protein which was reported to enhance plant drought resistance by increasing the levels of osmotic adjustment substances [56].

The strong positive correlations between Zn, Ca, Fe, and Se, the co-localization of their QTLs, and the observed SNP associations (Fig. S5) could be explained by overlap in these elements’ uptake and transport mechanisms. Several studies have reported correlations between different minerals [59–63]. Genetic mapping in cereals to elucidate the genetic basis underlying these correlations has been attempted [64–66].

QTL co-localization and pleiotropy markers were observed among minerals, TKW, protein and DR_B (Fig. 6). Previous studies suggested that gene pleiotropy and QTL co-localization play a role in correlations among mineral uptake [59–61].

Our results confirm that a highly complex genetic network at multiple loci controls grain nutrition levels [67–69]. The co-localization of QTLs for some traits indicate that simultaneous improvement of these traits may be possible in sorghum grain. Ca and Zn should be targeted for their potential linkage to DR, PH and TKW.

P shared a common QTL with Zn and Mg but not Fe in all accessions on Chr. 3 (Fig. 6a). Thus, selection at this locus to increase the concentrations of Zn and Mg is likely to increase the concentration of P. However, in mature grain, P is mainly stored as phytate, which can bind with Fe, forming insoluble complexes that humans cannot digest or absorb [70].

QTLs for Zn, Mn, other elements, TKW, PH and drought traits were found on Chrs. 1, 2, 4, 8, 9 and 10 in analyses of all accessions and on Chrs. 1, 5, 6, 8, 9 and 10 in analyses of African accessions (Fig. 6a, b). Previous studies have also reported that these regions are associated with several grain element concentrations, TKW, and grain yield [46, 71]. A computer-based analysis identified 77 candidate genes for Fe and Zn homeostasis; Chr. 1 had the highest number of genes (24) and Chr. 8 the least [69].

Overall, the genomic regions on Chrs. 1, 2, 4, 8, 9, and 10 that were associated with multiple traits could be used in attempts at simultaneous improvement of multiple nutrient concentrations, TKW, PH, and DR in sorghum breeding. Mapping of QTLs for TKW from African accessions to five chromosomal regions suggests that selection for higher grain nutrition and TKW may not incur a yield penalty. Several other studies have found strong correlations and shared QTLs between protein, fat, and starch, and between these traits and grain yield [17, 72–74]. Shared genetic control or developmental mechanisms of these grain components may underlie the correlations; however, some may be due to evolutionary correlations rather than a shared genetic or developmental basis. Further studies could help identify genes that control each of these traits.

Relationships between nutrient concentrations in banana depend on plant performance [75]. Most of the mineral traits potentially associated with DR traits in this study have a specific role in mitigating the negative effects of drought stress. Seed concentrations of K, P, Na, and B were higher in slow-wilting soybean genotypes under drought stress and may contribute to slow wilting by maintaining homeostasis and osmotic regulation [76]. The increase in osmoregulatory elements such as minerals could be beneficial for sorghum breeders in selecting for drought stress tolerance. Si has a role in enhancing plant biomass accumulation and reducing water loss through transpiration [77]. Mo application enhances water use capability, antioxidative defense, and osmotic adjustment under drought stress [78]. Zn is an essential micronutrient that plays a fundamental role in crop resistance to drought stress by regulating various physiological and molecular mechanisms. Under drought stress, Zn application improves seed germination, plant water relations, cell membrane stability, osmolyte accumulation, stomatal regulation, water use efficiency, and photosynthesis, resulting in significantly better plant performance [79]. Ca is an essential element for plants, functioning as a second messenger in cells, and can influence aquaporins on membrane structures, helping plants resist many kinds of environmental stress [80]. Higher glutathione pools most likely mediate the beneficial effect of Ca in tolerance to drought-induced oxidative stress, increased levels of the free polyamine putrescine, and lower levels of the amino acid gamma-aminobutyric acid [81].

The nine accessions that recovered from drought stress at 3 and 5 WAI demonstrate the possibility of selecting drought-tolerant accessions on the basis of grain weight and nutritional potential (Table S7). For example, one of these accessions, WSC from Tanzania, had above-average levels of Ca and Na combined with high ability to adapt to drought. In wheat, a substantial increase in yield under drought has been achieved by selecting for high grain yield potential [82].

## Conclusions

We identified sorghum landraces that are promising sources of genetic material for manipulation of grain composition, yield potential in terms of TKW and PH, and DR, as well as several loci associated with these traits. Our results will contribute to understanding the genetic basis of natural variation in sorghum grain nutrition, kernel weight potential, and DR. Further studies could explore loci associated with these traits for use in molecular breeding programs to modify sorghum grain composition and improve various traits. Quantifying alternative phenotypes, such as nutrient uptake from the soil and remobilization to the seed under drought and normal conditions, may increase breeding efficiency and contribute to improving nutritional quality and studied yield-related traits (TKW and PH) in drought-prone areas. We found that accessions from different regions possess different unique traits. Although we used an unequal number of accessions from different geographical regions, these results could be a trigger for more detailed studies to map the different traits to different geographical regions which can strongly help and impact the breeding programs. Moreover, despite the concentration on African sorghum germplasm in breeding programs, the Asian accessions showed interesting variability not existing in the African germplasm that can impact sorghum breeding.

To the best of our knowledge, this is the first attempt to identify the phenotypic and genotypic associations between sorghum grain weight and nutritional potential and DR traits. Our results indicate that it may be possible to select for DR without field testing by selecting for high grain mineral contents (Ca, Zn, Si, P, K, B and Na) and yield potential. Although additional validation is necessary, this method can be used as a preliminary selection strategy in breeding programs.

### Methods

#### Plant materials

The 163 sorghum (*Sorghum bicolor*) accessions used here have been deposited in the gene bank of the Arid Land Research Center, Tottori University, Japan (Supplementary Table S2). Of these, 105 were obtained from the National Agriculture and Food Research Organization’s gene bank, and their passport data and information can be acquired at https://www.gene.affrc.go.jp/about_en.php. The remaining 58 accessions were obtained from the Agricultural Plant Genetic Resources Conservation & Research Gene Bank in Sudan. Of these, 49 are landraces and 9 are cultivars. The 163 accessions were chosen to capture the broadest possible geographic distribution of sorghum globally, particularly from Asia and Africa. Of the Asian accessions, 23 came from East Asia (Japan, Korea, Taiwan, and China), 2 from Southeast Asia (Cambodia and Myanmar), 26 from South Asia (India, Pakistan, Afghanistan, Bangladesh, and Nepal), and 2 from Southwest Asia (Iran and Israel). The remaining African accessions came from North Africa (75 accessions from Sudan and Morocco), Central Africa ( 4 accessions from Chad and the Central African Republic), East Africa (13 accessions from Uganda, Ethiopia, Kenya and Tanzania), West Africa (5 accessions from Nigeria) and South Africa ( 13 accessions from Lesotho, South Africa and Zimbabwe).

### Drought tolerance evaluation

We evaluated the accessions in a glasshouse at the Arid Land Research Center. All accessions were grown in pots (23 cm x 18.5 cm, 6 L); each pot contained three replicates of each accession. All pots were filled with Tottori sand dune soil mixed with fertilizers (organic lime 5 g/pot; Hitachi Ace (HITACHI CHEMICAL INDUSTRIES Co., Ltd., Japan, Mg:14%, Mn:0.3%, B:0.3%)) 5 g/pot; NPK fertilizer 14–14–14, 5 g/pot). A corrugated sheet was laid under the bottom of the pots, and the roots were restricted so they could not emerge from the pot. Seeds were sown in the first week of June in 2017 and 2018. In the drought treatment, pots were irrigated normally until heading, then irrigation was stopped for 10 days. In the first year and during these 10 days of drought, we observed the plant senescence rate and scored it on a 6-point scale (5 good, 4 withered, 3 considerably withered but leaves are green, 2 considerably withered and senescence on some leaves, 1 considerably withered and senescence on many leaves, and 0 complete senescence). The withering index was measured at 4 (WI_4DAI), 6 (WI_6DAI), and 10 (WI_10DAI) days after irrigation in 2017 and at 4 (DR_A) and 10 (DR_B) in 2018 (Fig. S6). In both years different person scored the plants under drought and therefore the data were treated separately. We measured the leaf temperature of the highest unshaded leaf around noon at 0 (TM_0DAI), 4 (TM_4DAI), 6 (TM_6DAI), and 10 (TM_10DAI) days after drought treatment with a radiation thermometer (Thermometer Infrared Thermometer IT-545, HORIBA, Ltd., Japan). The TM_Index was calculated as a % increase in leaf temperature after 10 days.

Plant height (PH) was measured in cm at the heading. After the drought treatment, we re-irrigated the pots normally and observed the recovery from drought at 3 and 5 weeks. From the normally irrigated pots, without drought in season 2017, grains were harvested, TKW was measured in g, and seeds were used for mineral and protein content analysis.

### Protein content

Matured seeds were collected from irrigated plants, and stored at the desiccator for drying before analysis. Seeds were then ground for measurement of the N content of about 50 mg of sorghum kernel powder with a CN Corder (Model MT-700; Yanaco, Inc., Kyoto, Japan). We recorded the N: C ratio, then calculated the N content as a percentage, and converted that value to the crude protein content by multiplying the N content by 5.95 [83].

### Mineral quantification using inductively coupled plasma mass spectrometry analysis

Macro- (P, K, Mg, Ca, and Na) and micro- (Al, B, Ba, Cu, Fe, Si, Se, Mo, Mn, Zn, and S) mineral contents were quantified. About 200 mg of mature seeds was digested with concentrated HNO_3_ in a high-performance microwave digestion system (ETHOS UP, Milestone (MG), Kawasaki, Japan; http://www.milestonesrl.com). Digested samples were diluted 1:1000 before injection into an inductively coupled plasma-mass spectrometry (ICP-MS) system (Agilent 7700 ICP-MS, Agilent Technologies, Santa Clara, CA, USA).

### Statistical analysis

Data were tested for normality and homogeneity of variance before analysis by the Shapiro–Wilk test and Levene’s test, respectively. Analysis of variance (ANOVA) and mean comparison (Tukey’s test) were performed in GenStat software, 18th edition (VSNi, Hemel Hempstead, UK). A combined analysis of variance was carried out across 2 years and best linear unbiased prediction (BLUP) was used in subsequent analysis.

Morphological data for all 163 accessions were clustered by using the between-groups linkage method of squared Euclidean distance in SPSS v. 25 software (IBM, Chicago, IL, USA). Hierarchical cluster analysis based on average values of all traits and dendrogram visualization of the results in hierarchical cluster analysis was visualized in R program, 2018 [84].

Genetic distances were estimated by using Nei’s distance [85], and phylogenetic trees were based on the neighbor-joining method in Power Marker v. 3.25 software ([86], http://statgen.ncsu.edu/powermarker/). Branch support values were determined by performing bootstrap analysis with 1000 replicates. Power Marker was used to calculate the allelic number (NA), genetic diversity, and polymorphism information content (PIC) values of each marker. Population structures were evaluated in the R program v. 2018 [84]. Principal components (PCs) were calculated in GAPIT software as described by Wang and Zhang [87]; the first two PCs were plotted in R [84]. Analysis of molecular variance (AMOVA) and principal component analysis (PCA) of all inferred groups based on a genetic distance matrix were calculated in GenAlEx v. 6.5 software [88].

To maximize the differences and to detect those differences in all traits among groups, the current research applied a multivariate method, Visual Infusion Phlebitis (VIP) scores was performed based on the Partial Least Squares Discriminant Analysis (PLS-DA) using Metaboanalyst (ver.5) software.

### DNA extraction and genotyping by sequencing

DNA from the leaf tissue of 2-week-old seedlings of each line was extracted by using a modified CTAB method [89]. Samples were genotyped on the DArTseq platform (Diversity Arrays Technology, Australia), which represents a combination of DArT complexity reduction methods based on next-generation sequencing platforms [90].

### Genome‑wide association study

Drought data sets, seed weight, and nutrition-related traits were used to identify SNPs associated with all traits. GWAS analysis was performed separately on 158 (had good quality marker data) of the 163 accessions and on the 110 African accessions using 8938 SNP markers with < 20% missing data and ≤ 0.05 minor allele frequency. GWAS was performed in R software for multi-locus GWAS, namely mrMLM version 4.0.2 [32, 84]. The kinship matrix and top three principal components were used in the GWAS analysis to control population relatedness and geographical area structure with two different algorithms, mrMLM and the fast multi-locus random-mixed linear model (FASTmrMLM), which allowed us to overcome restrictions on the number of markers and to increase the statistical power [91]. The distribution of observed versus expected log_10_(*P*) values was visualized on quantile–quantile (Q–Q) plots to test the fitness of GWAS models for all traits [92], and the GWAS results were visualized on Manhattan plots. Significant marker-trait associations corresponding to putative QTLs for all analyzed traits were determined at LOD value ≥ 3 as recommended by [32, 93].

To summarize all the GWAS results, significant marker-trait associations were plotted in PhenoGram software (Biomedical and Translational Informatics Laboratory: https://ritchielab.org/software/phenogram). Manhattan and Q–Q plots were plotted in R.

### Electronic supplementary material

Below is the link to the electronic supplementary material.

Supplementary Material 1

Supplementary Material 2

## Data Availability

All data supporting the findings of this study are available within the paper and its Supplementary Information. Genotyping data is available upon request from the corresponding author. Genotyping data is stored at the gene bank of the Arid Land Research Center, Tottori University. All accessions used in this study are available through the gene bank of the Arid Land Research Center, Tottori University, Japan.

## Abbreviations

GWAS: Genome-wide association study
DR: Drought resistance (drought tolerance)
TKW: Thousand-kernel weight
PH: Plant height
QTL: Quantitative trait locus
WI_4DAI, WI_6DAI, WI_10DAI: Withering index measured at 4, 6, and 10 days after irrigation
TM_0DAI, TM_4DAI, TM_10DAI: Leaf temperature at 0, 4, 6, and 10 days after drought treatment
TM_Index: % increase of leaf temperature after 10 days of drought
VIP: Visual Infusion phlebitis
PLSD: Partial Least Squares Discriminant Analysis
DArT: Diversity array technology
mrMLM: Multi-locus random SNP effect-mixed linear model
FASTmrMLM: Fast multi-locus random-SNP effect mixed linear model
SNP: Single nucleotide polymorphism
Q–Q: Quantile–quantile
PCA: Principal component analysis
