# Supplementary material for: Genetic variation for grain nutritional profile and yield potential in sorghum and the possibility of selection for drought tolerance under irrigated conditions
Source: BMC Genomics. 2023 Sep 2;24:515. doi: 10.1186/s12864-023-09613-w (PMC10474746; doi:10.1186/s12864-023-09613-w)
Supplement: Supplementary file 1 — Supplementary Material 1 [file 12864_2023_9613_MOESM1_ESM.pdf]

(a)

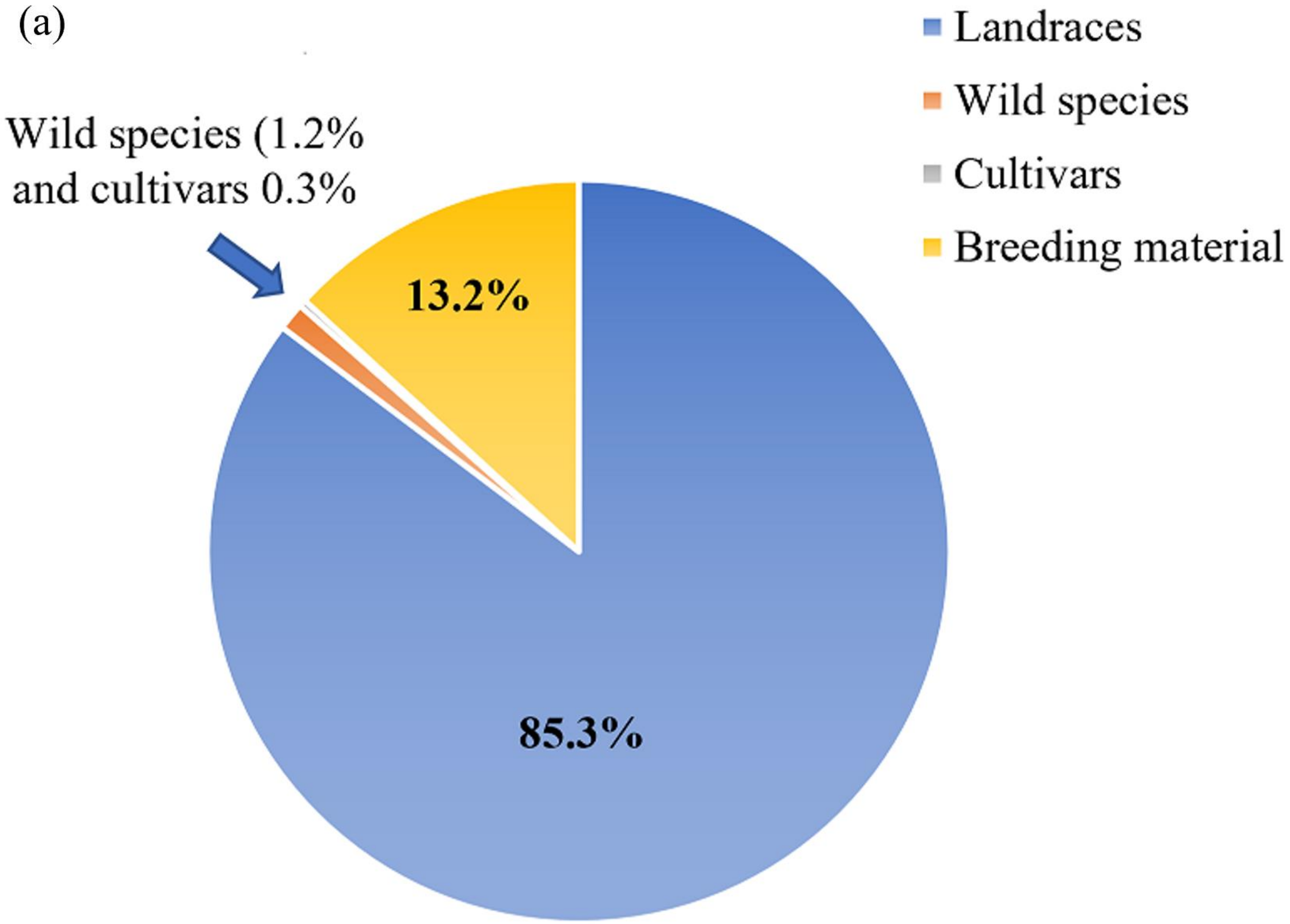

(b)

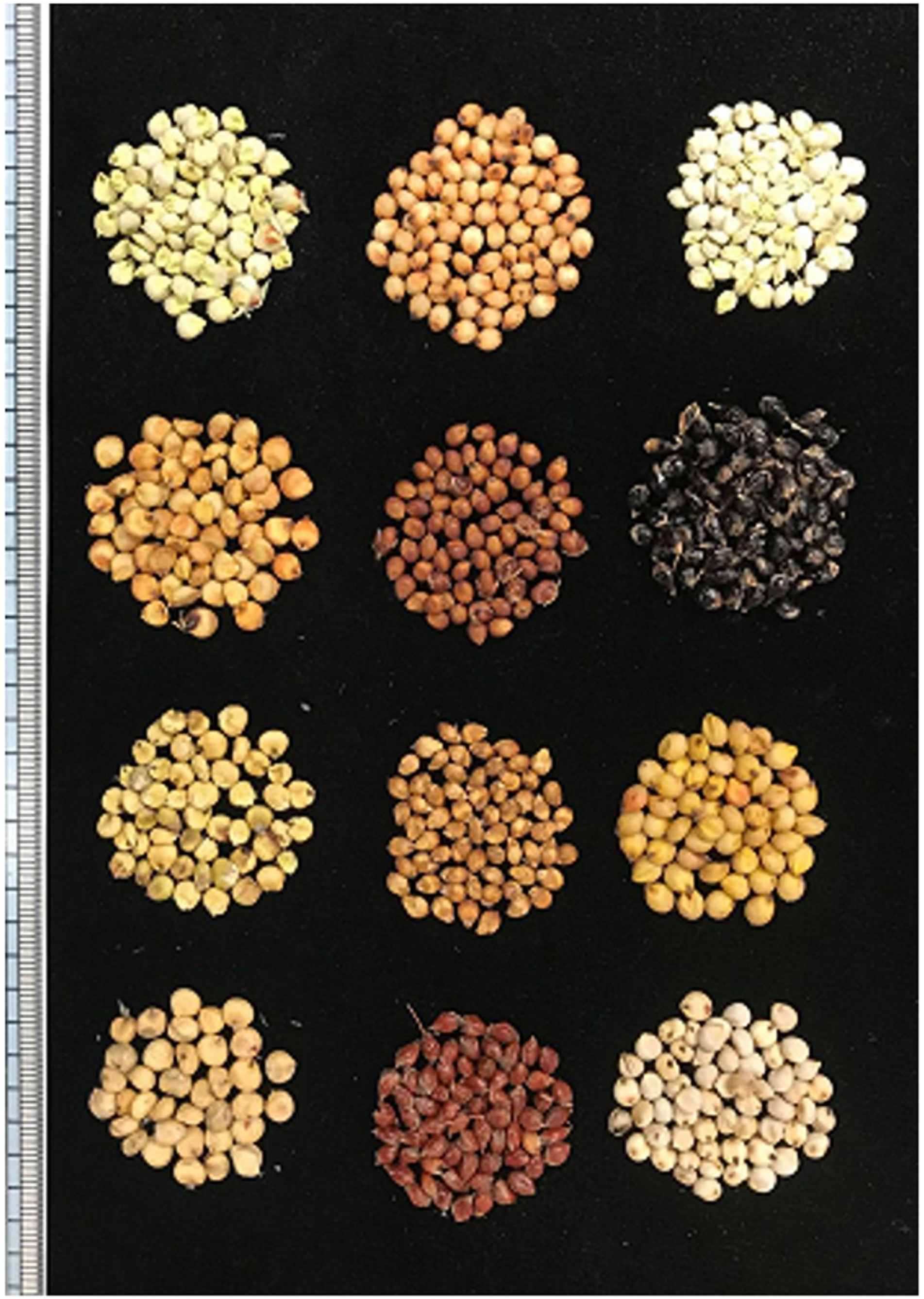

Additional file 1: Figure S1. (a) The genetic pool used in sorghum breeding is very small; adopted from ICRISAT: http://icrisat.org/crop-sorghum-genebank.hto. (b) Representative samples of sorghum genetic diversity from our study.

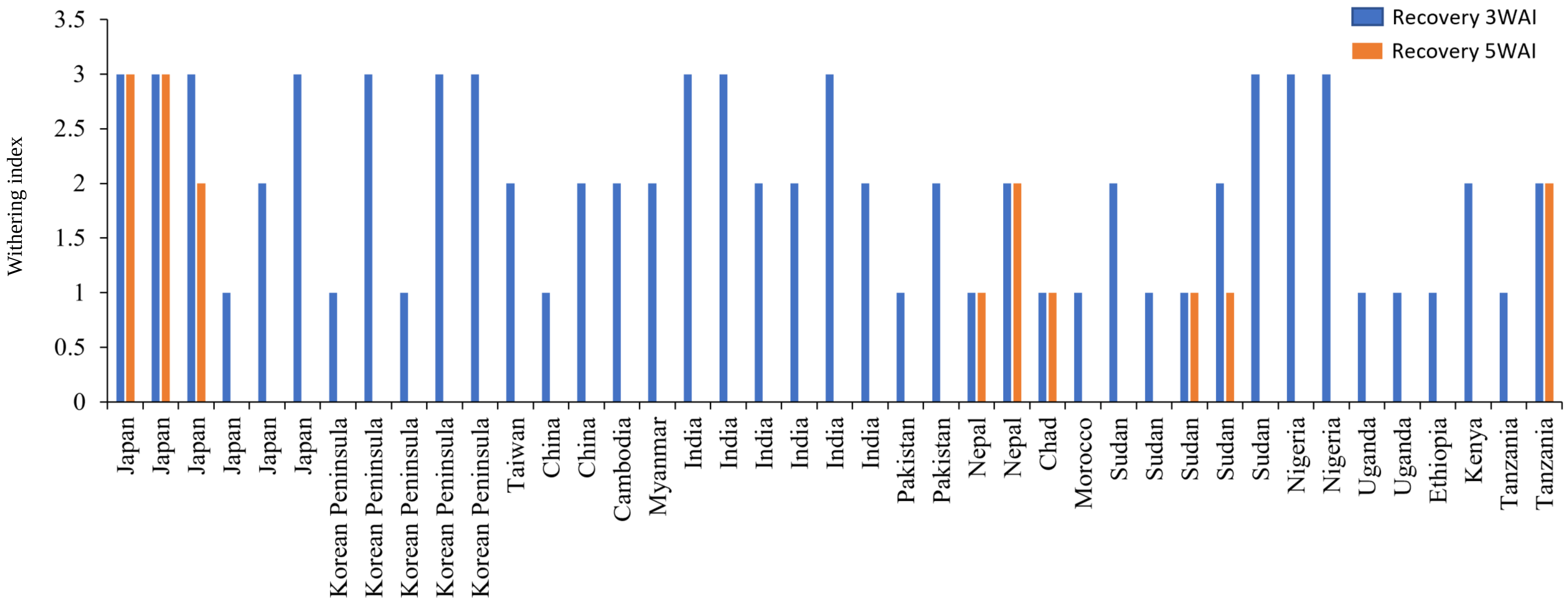

Additional file 1: Figure S2. Withering index of forty-one Asian and African accessions recovered from drought after 3 and 5 weeks after re-irrigation.

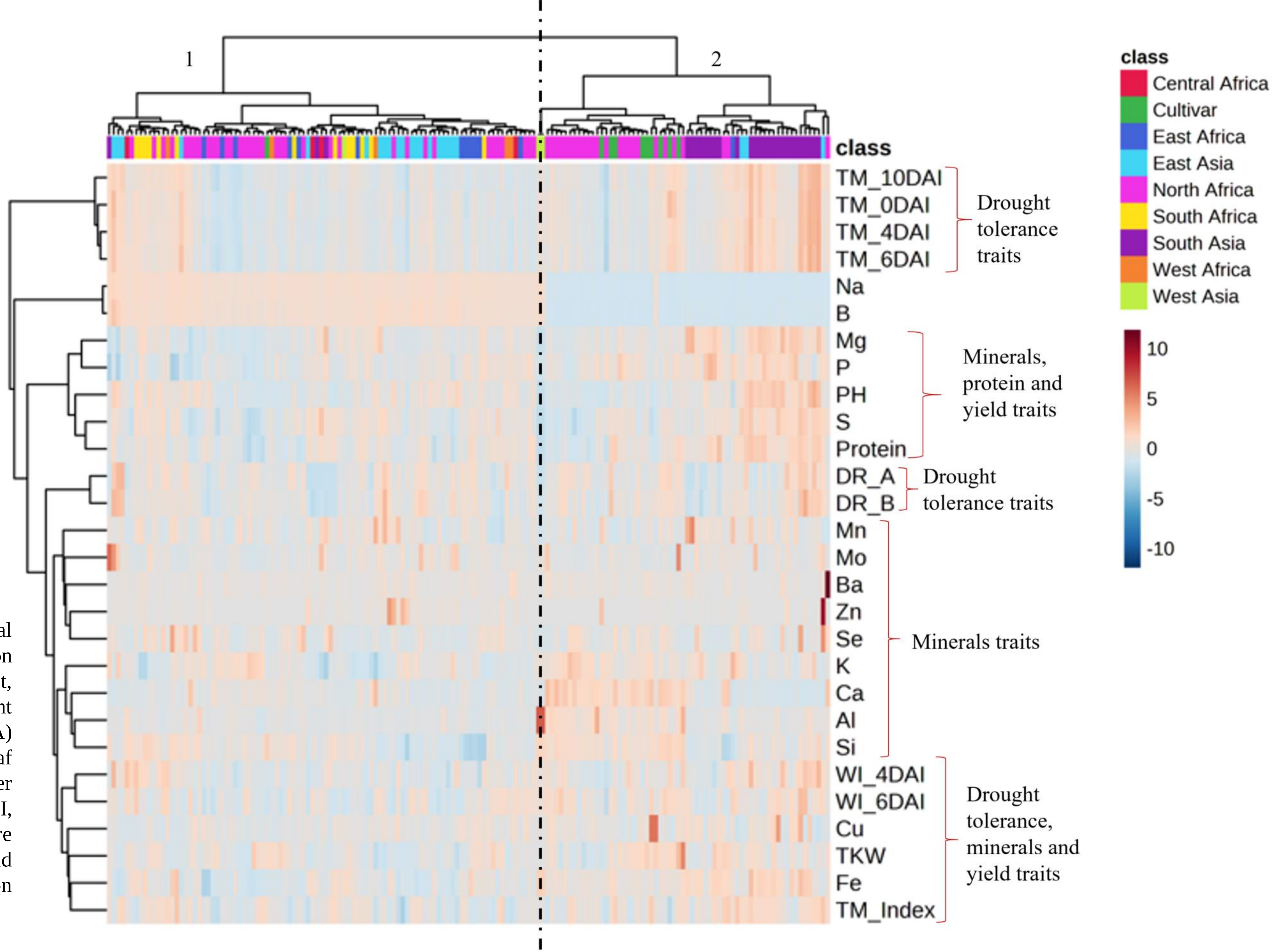

Additional file 1: Figure S3. Hierarchical clustering of the 163 sorghum accessions on the basis of 16 grain elements, protein content, thousand kernel weight (TKW), plant height (PH), drought resistance score at 4 (DR_A) and 6 days after irrigation (DR_B), leaf temperature at 0, 4, 6, and 10 days after irrigation (TM_0DAI, TM_4DAI, TM_6DAI, TM_10DAI), % increase of leaf temperature after 10 days of drought (TM_Index), and withering index at 4 and 6 days after irrigation (WI 4DAI, WI 6DAI).

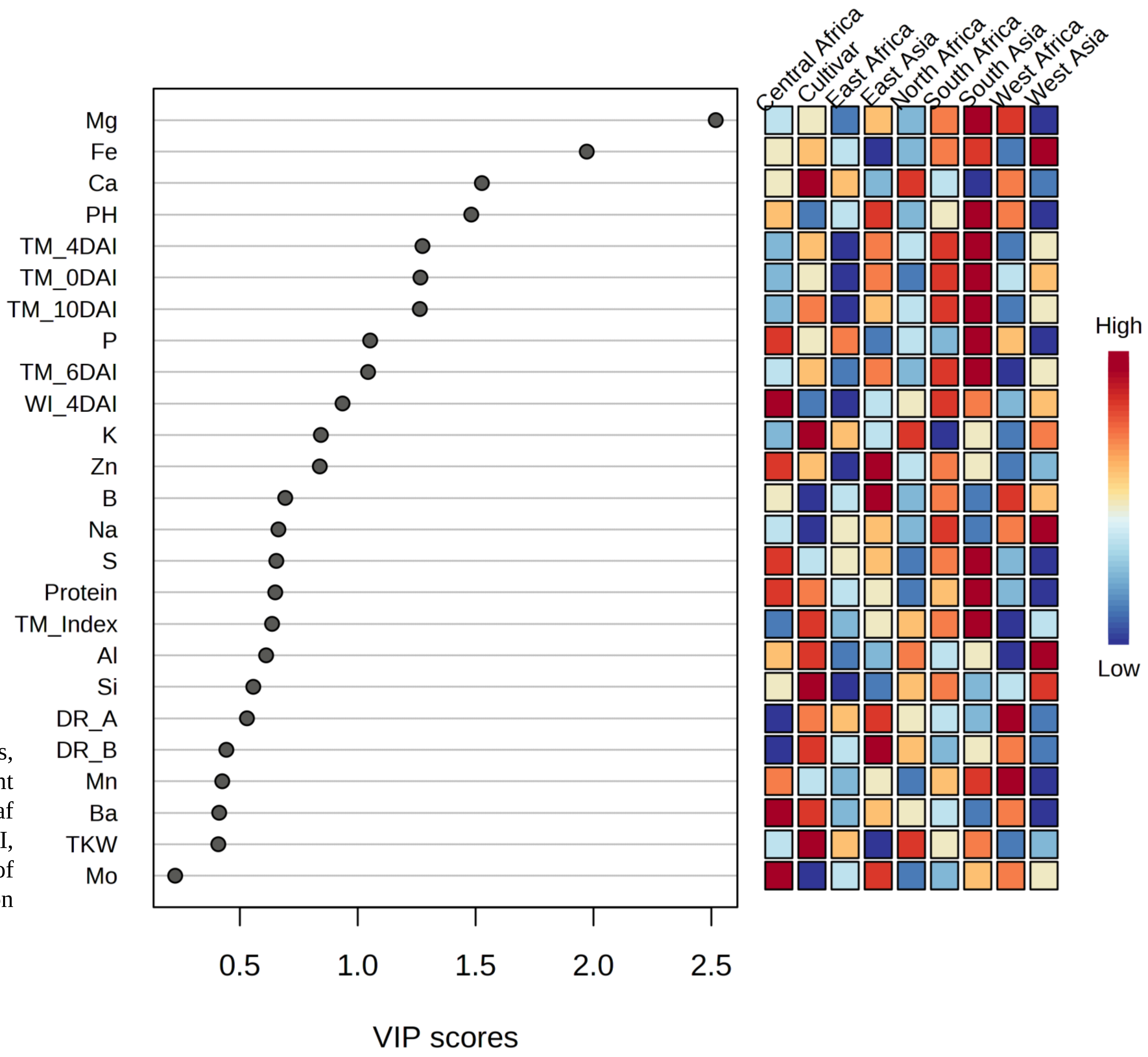

Additional file 1: Figure S4. VIP scores on the basis of 16 grain elements, protein content, thousand kernel weight (TKW), plant height (PH), drought resistance score at 4 (DR_A) and 6 days after irrigation (DR_B), leaf temperature at 0, 4, 6, and 10 days after irrigation (TM_0DAI, TM_4DAI, TM_6DAI, TM_10DAI), % increase of leaf temperature after 10 days of drought (TM_Index), and withering index at 4 and 6 days after irrigation (WI 4DAI, WI 6DAI).

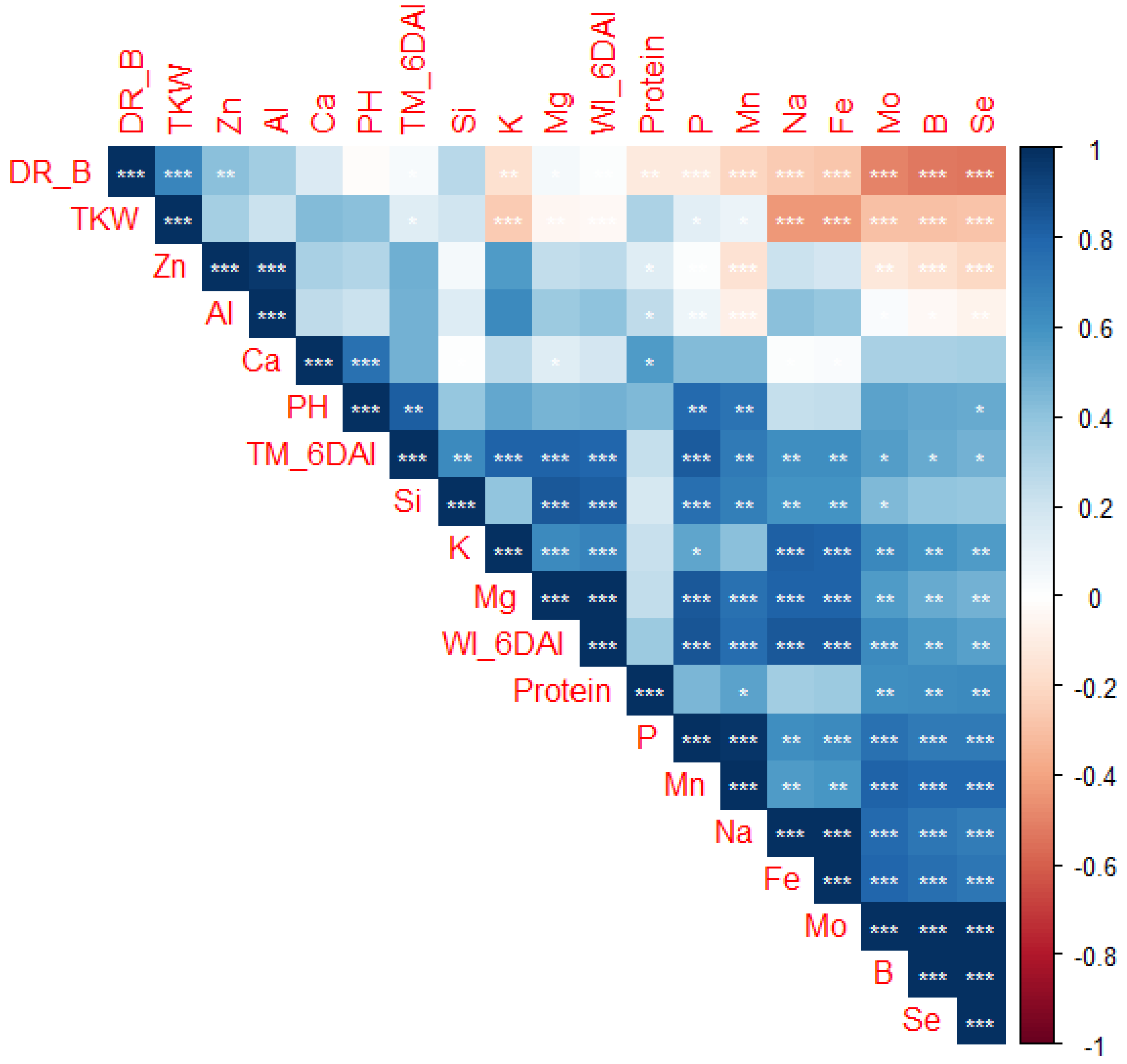

Additional file 1: Figure S5. Pairwise statistical associations among traits' significant SNPs in all sorghum accessions.

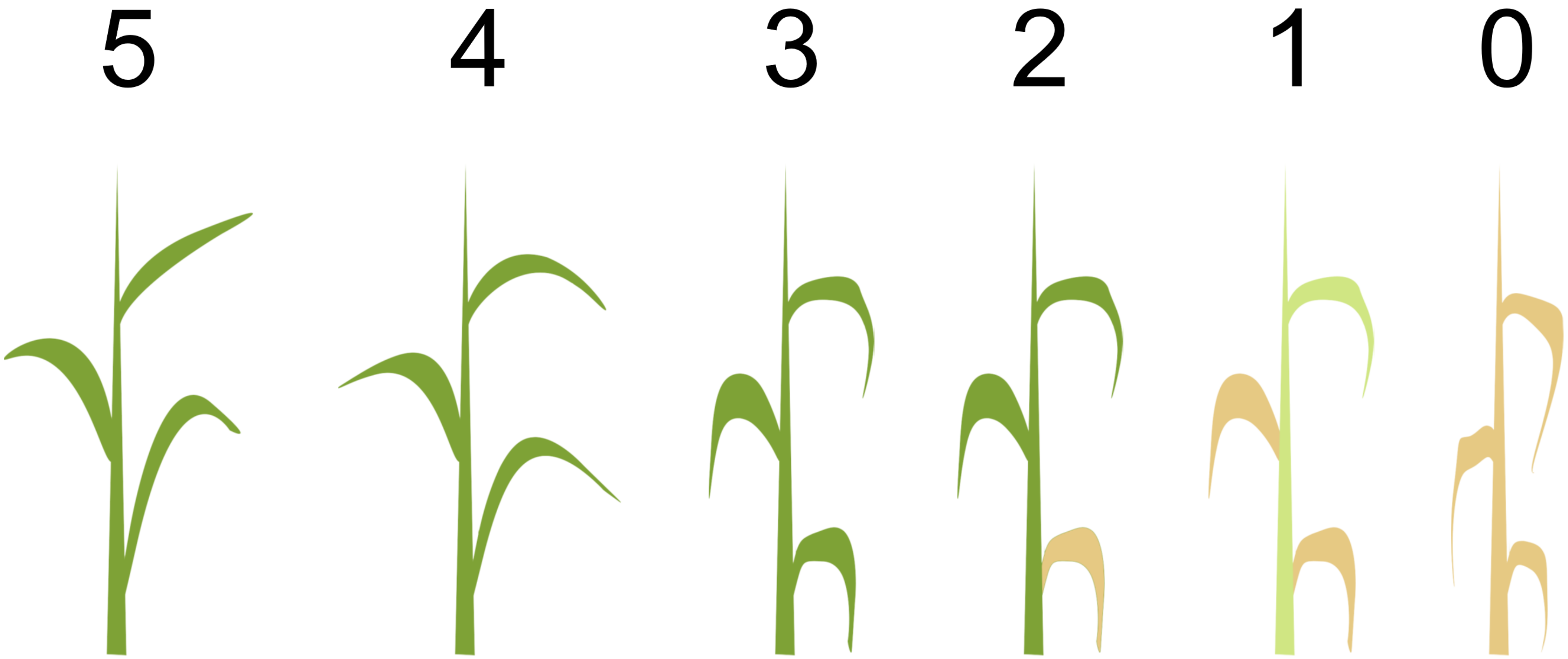

Additional file 1: Figure S6. The illustration explains scale points used to score sorghum accessions for drought response.
